# Visualizing the role of applied voltage in non-metal electrocatalysts

**DOI:** 10.1093/nsr/nwad166

**Published:** 2023-06-06

**Authors:** Ziyuan Wang, Jun Chen, Chenwei Ni, Wei Nie, Dongfeng Li, Na Ta, Deyun Zhang, Yimeng Sun, Fusai Sun, Qian Li, Yuran Li, Ruotian Chen, Tiankai Bu, Fengtao Fan, Can Li

**Affiliations:** Collaborative Innovation Center of Chemistry for Energy Materials (iChEM), State Key Laboratory of Catalysis, Dalian National Laboratory for Clean Energy, Dalian Institute of Chemical Physics, Chinese Academy of Sciences, Dalian 116023, China; Department of Chemistry, Collaborative Innovation Center of Chemistry for Energy Materials (iChEM), College of Chemistry and Chemical Engineering, Xiamen University, Xiamen 361005, China; Collaborative Innovation Center of Chemistry for Energy Materials (iChEM), State Key Laboratory of Catalysis, Dalian National Laboratory for Clean Energy, Dalian Institute of Chemical Physics, Chinese Academy of Sciences, Dalian 116023, China; Energy College, University of Chinese Academy of Sciences, Beijing 100049, China; Collaborative Innovation Center of Chemistry for Energy Materials (iChEM), State Key Laboratory of Catalysis, Dalian National Laboratory for Clean Energy, Dalian Institute of Chemical Physics, Chinese Academy of Sciences, Dalian 116023, China; Energy College, University of Chinese Academy of Sciences, Beijing 100049, China; Collaborative Innovation Center of Chemistry for Energy Materials (iChEM), State Key Laboratory of Catalysis, Dalian National Laboratory for Clean Energy, Dalian Institute of Chemical Physics, Chinese Academy of Sciences, Dalian 116023, China; Energy College, University of Chinese Academy of Sciences, Beijing 100049, China; Collaborative Innovation Center of Chemistry for Energy Materials (iChEM), State Key Laboratory of Catalysis, Dalian National Laboratory for Clean Energy, Dalian Institute of Chemical Physics, Chinese Academy of Sciences, Dalian 116023, China; Energy College, University of Chinese Academy of Sciences, Beijing 100049, China; Collaborative Innovation Center of Chemistry for Energy Materials (iChEM), State Key Laboratory of Catalysis, Dalian National Laboratory for Clean Energy, Dalian Institute of Chemical Physics, Chinese Academy of Sciences, Dalian 116023, China; Collaborative Innovation Center of Chemistry for Energy Materials (iChEM), State Key Laboratory of Catalysis, Dalian National Laboratory for Clean Energy, Dalian Institute of Chemical Physics, Chinese Academy of Sciences, Dalian 116023, China; Energy College, University of Chinese Academy of Sciences, Beijing 100049, China; Collaborative Innovation Center of Chemistry for Energy Materials (iChEM), State Key Laboratory of Catalysis, Dalian National Laboratory for Clean Energy, Dalian Institute of Chemical Physics, Chinese Academy of Sciences, Dalian 116023, China; Energy College, University of Chinese Academy of Sciences, Beijing 100049, China; Collaborative Innovation Center of Chemistry for Energy Materials (iChEM), State Key Laboratory of Catalysis, Dalian National Laboratory for Clean Energy, Dalian Institute of Chemical Physics, Chinese Academy of Sciences, Dalian 116023, China; Energy College, University of Chinese Academy of Sciences, Beijing 100049, China; Collaborative Innovation Center of Chemistry for Energy Materials (iChEM), State Key Laboratory of Catalysis, Dalian National Laboratory for Clean Energy, Dalian Institute of Chemical Physics, Chinese Academy of Sciences, Dalian 116023, China; Energy College, University of Chinese Academy of Sciences, Beijing 100049, China; Collaborative Innovation Center of Chemistry for Energy Materials (iChEM), State Key Laboratory of Catalysis, Dalian National Laboratory for Clean Energy, Dalian Institute of Chemical Physics, Chinese Academy of Sciences, Dalian 116023, China; Department of Chemistry, Collaborative Innovation Center of Chemistry for Energy Materials (iChEM), College of Chemistry and Chemical Engineering, Xiamen University, Xiamen 361005, China; Collaborative Innovation Center of Chemistry for Energy Materials (iChEM), State Key Laboratory of Catalysis, Dalian National Laboratory for Clean Energy, Dalian Institute of Chemical Physics, Chinese Academy of Sciences, Dalian 116023, China; Department of Materials, Imperial College London, London SW7 2AZ, UK; Collaborative Innovation Center of Chemistry for Energy Materials (iChEM), State Key Laboratory of Catalysis, Dalian National Laboratory for Clean Energy, Dalian Institute of Chemical Physics, Chinese Academy of Sciences, Dalian 116023, China; Collaborative Innovation Center of Chemistry for Energy Materials (iChEM), State Key Laboratory of Catalysis, Dalian National Laboratory for Clean Energy, Dalian Institute of Chemical Physics, Chinese Academy of Sciences, Dalian 116023, China

**Keywords:** non-metal electrocatalyst, surface potential, electron transfer, hydrogen evolution reaction, mapping

## Abstract

Understanding how applied voltage drives the electrocatalytic reaction at the nanoscale is a fundamental scientific problem, particularly in non-metallic electrocatalysts, due to their low intrinsic carrier concentration. Herein, using monolayer molybdenum disulfide (MoS_2_) as a model system of non-metallic catalyst, the potential drops across the basal plane of MoS_2_ (ΔV_sem_) and the electric double layer (ΔV_edl_) are decoupled quantitatively as a function of applied voltage through *in-situ* surface potential microscopy. We visualize the evolution of the band structure under liquid conditions and clarify the process of E_F_ keeping moving deep into E_c_, revealing the formation process of the electrolyte gating effect. Additionally, electron transfer (ET) imaging reveals that the basal plane exhibits high ET activity, consistent with the results of surface potential measurements. The potential-dependent behavior of k_f_ and n_s_ in the ET reaction are further decoupled based on the measurements of ΔV_sem_ and ΔV_edl_. Comparing the ET and hydrogen evolution reaction imaging results suggests that the low electrocatalytic activity of the basal plane is mainly due to the absence of active sites, rather than its electron transfer ability. This study fills an experimental gap in exploring driving forces for electrocatalysis at the nanoscale and addresses the long-standing issue of the inability to decouple charge transfer from catalytic processes.

## Teaser

By measuring the MoS_2_'s surface potential under various applied potentials, we observe the insulator-to-conductor transition in the basal plane and establish a significant connection to spatially mapped electrocatalytic activity.

## INTRODUCTION

Electrochemical reactions are a key strategy for realizing carbon neutrality and advancing the utilization of clean energy through the production of green hydrogen via water electrolysis with renewable electricity. Unraveling the electrocatalytic mechanism is of paramount importance to achieve a comprehensive understanding of the process and develop high-performance catalysts. Therefore, investigating the electrocatalytic mechanism holds significant promise for promoting a sustainable future. Applying voltage is an effective method for changing the electrochemical potential of an electrocatalyst [1,2], thereby determining the direction and rate of the reaction. However, the applied voltage (V_appl_) regulates the electrochemical process differently for metal and non-metal catalysts. For metal catalysts with a huge density of states (DOS) near the Fermi levels (E_F_), the potential mainly drops within the electric double layer (EDL), modulating the electrostatic potential (φ) and changing the reaction coordinates directly [1]. While for non-metallic catalysts, due to the low electrical conductivity and complex changes in chemical composition, the potential distribution across the solid–liquid interface becomes complicated [3–8]. A part of the potential drop may drop across the non-metallic catalysts, while another part of the potential drops across the EDL [6–8]. In this case, both the chemical potential (μ_e_) and the electrostatic potential (φ) are changed, resulting in the complex electrocatalytic mechanism. Due to the wide variety of non-metal electrocatalysts, finding the universal rules is a great challenge. For example, it was reported that electrocatalytic oxygen evolution reaction (OER) is driven by the chemical potential (μ_e_) in an IrO_x_ catalyst, rather than its electrostatic potential [2,9]. While for M–N-doped carbon catalysts, the electrocatalytic reaction is driven predominantly by the change in electrostatic potential across the EDL [2,10,11]. If the potential distribution across the solid–liquid interface can be directly measured, it will be very convenient for studying the electrocatalytic mechanism of non-metallic catalysts.

Up until now, distinguishing the potential distribution between the non-metallic catalyst and the EDL has still relied on complex theoretical calculations [3,4,12]. By using empirical formulas of capacitance, Bediako *et al.* showed that the potential distribution between twisted bilayer graphene and EDL is a function of *V_appl_* [8]. In the field of conventional electrochemical technology, Mott-Schottky analysis is a common method for calculating the flat band potential of semiconductors. However, it cannot provide information on how the band structure moves, which is related to the interfacial potential distribution. Through *I-V* curve, the onset potential of a low-DOS semiconductor at which the E_F_ reaches the band edge can be obtained [13]. However, the potential drop across the semiconductor–electrolyte interface still remains unknown, and hence it is not possible to obtain the respective shift of E_c_ and E_F_. The value of the total interface capacitance (*C_i_*) can be obtained by electrochemical impedance spectroscopy (EIS). However, the specific capacitance of the non-metallic electrode and EDL cannot be directly measured by EIS, which requires calculations based on the non-metallic structure model and the double layer (Gouy–Chapman-Stern) model, respectively [6]. In summary, there is a lack of *in-situ* techniques for directly measuring the potential distribution between the non-metallic catalyst and the EDL. Moreover, conventional electrochemical characterization only provides the ensemble information for electrode materials, neglecting the spatial heterogeneity in the electronic structures of catalysts [14–20]. Therefore, a spatially resolved *in-situ* characterization technique is needed.

Scanning electrochemical potential microscopy (SECPM), invented by Bard's group, has been used to measure EDL profiles on electrode surfaces, demonstrating the feasibility of scanning probe techniques for potential measurements in liquid [21]. Boettcher *et al.* developed a contact-based potential-sensing electrochemical atomic force microscopy technique and successfully mapped the photovoltage at nanoscopic semiconductor–catalyst interfaces [22,23]. This shows that changes in surface potential of catalysts can be sensed through contact mode. Based on these pioneering works, we focus on the key issues in electrocatalysis, to reveal the working principle of electrocatalysts through *in-situ* surface potential microscopy. Recently, the spatial heterogeneity of local electrostatic potential at Au nanoplate–solution interfaces was revealed under open circuit potential (OCP) conditions [24]. However, it is still a challenge to figure out how the electronic structure of non-metallic electrocatalysts changes under applied voltage.

Semiconductors have been predicted to be non-ideal catalysts due to their low intrinsic carrier concentration [25]. For example, it is widely believed that the basal plane of MoS_2_ impedes charge transfer in electrocatalytic reactions [26–31]. According to classical electron transfer theories, the Schottky-analogue junction is broken and becomes ohmic once the E_F_ is tuned into the bands, resulting in the low conductivity of the semiconductor in liquid [13,32]. However, it is difficult to explain the high electrocatalytic activities of some semiconductor catalysts [13,33,34]. Liu *et al.* found that the ultrathin MoS_2_ can be modulated to be highly conductive (‘on’) or insulating (‘off’), strongly correlating with the hydrogen evolution reaction (HER) [13]. Morpurgo's group also observed liquid-gating-induced superconductivity in thin MoS_2_ crystals [35]. The Y_1.75_Co_0.25_Ru_2_O_7−δ_ electrocatalyst was found to enhance the charge transfer step in the oxygen evolution reaction (OER) [36], while the β-Co(OH)_2_ electrocatalyst exhibited gradually increasing conductivity after the onset potential of the OER [17]. The above results showed that a charge transport pathway can be open under solution conditions, reflecting the dramatic change of the energy band structure of the semiconductor. However, the underlying reason for the enhanced conductance is not clear.

MoS_2_ is a representative material in both the fields of electrocatalysis and semiconductor devices. Studying how the voltage affects the band structure and conductivity of MoS_2_ under solution conditions is of great research significance. Herein, using MoS_2_ as a non-metallic model system, the potential distribution between the basal plane of monolayer (ML) MoS_2_ (ΔV_sem_) and the electric double layer (ΔV_edl_) were quantificationally decoupled as a function of the applied voltage. We visualized how E_F_ moves into E_c_ of the basal plane of MoS_2_, thereby realizing the transition of conductivity, which is the origin of the enhanced conductance in liquid. We provide a method for studying electrochemical driving force at the nanoscale and demonstrate that only when the electron transfer site and the chemical site are spatially coincident can the energy conversion efficiency be maximized. This work has significant importance in advancing our understanding of the electrocatalytic mechanism, and provides theoretical guidance for the conversion of clean energy and the achievement of carbon neutrality.

## RESULTS AND DISCUSSION

### Decoupling the potential drops across the semiconductor (ΔV_sem_) and the electric double layer (ΔV_edl_)

**Figure 1.**
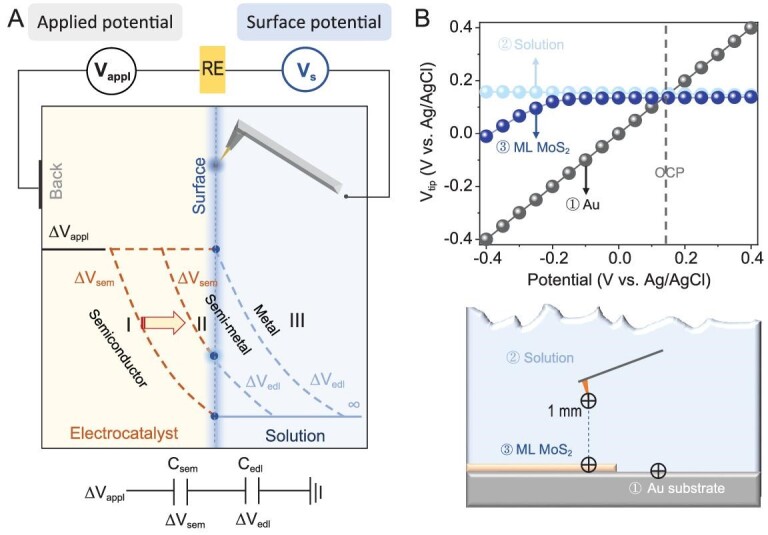
Local surface potential measurement on ML MoS_2_. (A) Schematic of *in-situ* surface potential measurement set-up and potential drops across the electrode–electrolyte interface for metal, semi-metal and semiconductor respectively. The potential of the tip (V_tip_) with respect to an Ag/AgCl reference electrode can be recorded through a high impedance amplifier with negligible leakage current. The potential distribution between catalyst (ΔV_sem_) and electrolyte (ΔV_edl_) is determined by the series of quantum capacitor (C_q_) and EDL capacitor (C_edl_), 1/C_tot_ = 1/C_q_ + 1/C_edl_. (B) Surface potential values of the basal plane of ML MoS_2_, Au and solution were measured with a tip in 0.1 M K_2_SO_4_. LSV was performed on the substrate (v = 10 mV/s, sample interval = 0.05 V), while the tip recorded the surface potential relative to the Ag/AgCl reference electrode.

Schematic illustrations of the home-built *in-situ* surface potential microscope are shown in Fig. 1A and Fig. S1. By combining a high impedance amplifier (1 TΩ) to the atomic force microscopy (AFM) positioning, the local surface potential V_s_ of the electrode can be obtained. This is achieved by reading the potential of the tip (V_tip_) in direct contact with the electrode surface relative to the Ag/AgCl reference based on an assumption: the E_F_ of the tip is controlled by the solution and not by the semiconductor in the ranges of applied voltage (the tip and the semiconductor are difficult to equilibrate, so the E_F_ of the tip does not follow the E_F_ of the semiconductor, and the potential of the tip (V_tip_) reflects the EDL potential at the surface of the semiconductor (V_s_)).

We take V_s_ (V_appl_ = 0) as a reference. The change of potential drop over the EDL (ΔV_edl_) can be read directly by V_s_ (V_appl_ ≠ 0)−V_s_ (V_appl_ = 0). By combining with the change of applied voltage (ΔV_appl_), the change of potential drop across the electrocatalyst (ΔV_sem_) can also be obtained by ΔV_sem_ = ΔV_appl_−ΔV_edl_. To eliminate the effect of redox reactions on the tip surface, which affect the potential measurements, we used a solution that contains only the supporting electrolytes (0.1 M K_2_SO_4_) and studied the intrinsic properties of ML MoS_2_ at different V_appl_.

Due to the small Debye screening length, the distance between the tip and the sample determines whether an accurate surface potential can be measured. To demonstrate the feasibility of surface potential measurement, a metal (Au) substrate was used as a standard sample. The tip was rested on the surface with an applied force of ∼29 nN, slightly larger than that reported by Boettcher *et al.* (∼25 nN) [21] but sufficient to prevent damage to the sample. As shown in Fig. 1B, V_s_ faithfully tracked V_appl_, reflecting that the surface potential V_s_ is equal to the applied voltage. This is consistent with the common view for bulk metal electrodes [1,37] that there is no potential drop inside the metal. In this situation, the surface electron concentration n_s_ can be considered a constant value and ΔV_appl_ is applied to increase the electric field intensity of EDL (ΔV_appl_ = ΔV_edl_), as indicated by curve III of Fig. 1A. With the same applied force (29 nN), the tip can be considered to be able to contact the surface of other samples. Then, the tip was lifted off ∼1 mm from the surface and the potential of the solution (background) was measured (Fig. 1B). It shows that the change in solution potential (∼12 mV) is negligible because the thickness of the EDL is less than 1 nm in a 0.1 M K_2_SO_4_ solution [38]. Therefore, the solution can be considered a blank background and does not affect the surface potential measurement.

**Figure 2.**
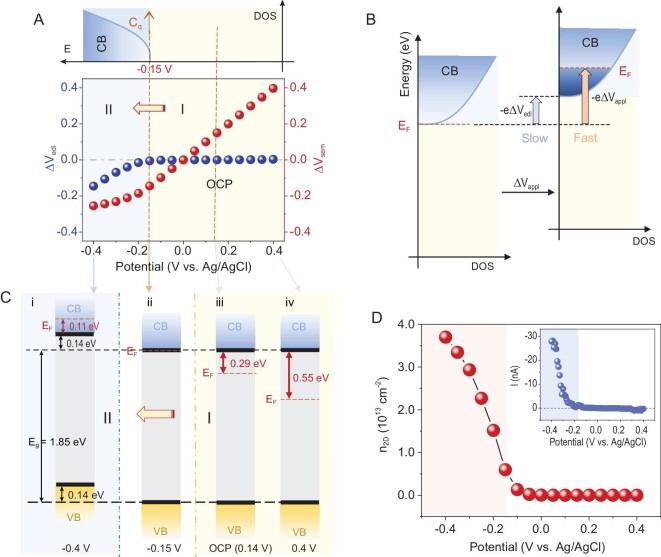
Decoupling of potential drops across MoS_2_ and EDL, and evolution of MoS_2_ band structure. (A) The decoupled contribution of each potential drop (ΔV_sem_ and ΔV_edl_) across the ML MoS_2_ basal plane–electrolyte interface as a function of V_appl_. ΔV_edl_ is obtained by V_s_ (V_appl_ ≠ 0)−V_s_ (V_appl_ = 0), while ΔV_sem_ can be obtained by ΔV_appl_−ΔV_edl_. A cartoon of the DOS of the ML MoS_2_ basal plane is shown in the top panel of (A). The E_F_ is tuned into the band edge of the conduction band (E_c_) at −0.15 V, leading to an increase in local density of states at the E_F_. (B) The change of applied voltage (ΔV_appl_) shifts the E_F_ of the basal plane by −eΔV_appl_ relative to its initial position. The potential change in EDL (ΔV_edl_) shifts the full band structure (including the conduction band (E_c_) and the valence band (E_v_)) by −eΔV_edl_ relative to its initial position. The potential change in the semiconductor (ΔV_sem_) corresponds to the relative movement rate between E_F_ and E_c_, shifting the E_F_ by −eΔV_sem_ with respect to the E_c_, which contributes to the change of surface conductance. (C) Energy diagrams for the basal plane of ML MoS_2_ at different V_appl_. (D) The n_s_-V_appl_ curve shows the switching effect of n_s_: the surface is turned ‘on’ with a high electron concentration under cathodic potential with an onset potential of approximately −0.15 V, while remaining insulated under anodic potential. Inset of (D), *in-situ* electric conductivity measurement on the basal plane of ML MoS_2_ in 0.1 M K_2_SO_4_. The AFM-SECM tip directly contacted the surface of the basal plane and recorded the conductivity current *I* (SG/TC mode, lift = 0, E_Tip_ = 0 V (vs. Ag/AgCl), LSV was performed on the substrate at v = 10 mV/s).

Next, the surface potential of the basal plane of ML MoS_2_ at different V_appl_ was monitored, as shown in Fig. 1B. Under OCP conditions (0.14 V), there was no apparent V_s_ change when the tip was transferred from the solution to the surface of MoS_2_. The result reflects that the solution has equilibrated the E_F_ of the tip and MoS_2_ before contact and the electron transfer between the tip and MoS_2_ can be ignored. The result demonstrates that the introduction of the nanotip did not change the initial structure of MoS_2_. When V_appl_ > −0.15 V (vs. Ag/AgCl), there is no change in V_s_. V_s_ starts to track V_appl_ when V_appl_ < −0.15 V. The change in EDL potential (ΔV_edl_) as a function of applied potential can be obtained by V_s_(V_appl_ ≠ 0) −V_s_ (V_appl_ = 0), as shown in Fig. 2A (blue circuit). Since a part of the potential drop over EDL is obtained, another part of the potential drop across the basal plane of MoS_2_ can also be given by ΔV_sem_ = ΔV_appl_−ΔV_edl_ (red circuit, Fig. 2A). The results clearly show that when V_appl_ > −0.15 V, the total potential is dropped across the ML MoS_2_ (ΔV_appl_ = ΔV_sem_), as shown in Fig. 1A, potential curve, type I, while the surface potential remains constant. When V_appl_ is below −0.15 V, the surface potential begins to change, resulting in a partial drop of the potential across the solution (ΔV_appl_ = ΔV_sem_ + ΔV_edl_, Fig. 1A, potential curve, type II). ΔV_edl_ increases as V_appl_ becomes more negative. After decoupling the potential drops in the basal plane (ΔV_sem_) and the EDL (ΔV_edl_), the evolution of the band structure under electrode conditions can be further revealed.

### The evolution of the band structure under electrode conditions

The evolution of the potential curve type I to type II reflects a dramatic change in band structure of the basal plane. As shown in Fig. 2B, the change in applied voltage (ΔV_appl_) shifts E_F_ of the basal plane by −eΔV_appl_ relative to its initial position [4,8], which is considered as the movement rate of E_F_ in the energy coordinates. The potential change in EDL (ΔV_edl_) shifts the full band structure (including the conduction band (E_c_) and the valence band (E_v_)) by −eΔV_edl_ relative to its initial position [1], which can be seen as the movement rate of E_c_. The potential change in the semiconductor (ΔV_sem_) corresponds to the relative rate difference between E_F_ and E_c_, shifting E_F_ by −eΔV_sem_ with respect to E_c_, which contributes to the change in surface conductance or n_s_ [4,8]. The potential distribution between the catalyst (ΔV_sem_) and the electrolyte (ΔV_edl_) is determined by the series of semiconductor capacitors (C_sem_) and EDL capacitors (C_edl_) as shown in Fig. 1 A (bottom). Additionally, due to the smaller DOS of 2D semiconductors compared to bulk semiconductors, 2D semiconductor capacitors function as quantum capacitors (Cq), where 1/C_tot_ = 1/C_q_ + 1/C_edl_ [5^6^,^39^].

When V_appl_ > −0.15 V, ΔV_appl_ = −ΔE_F_/e and ΔV_edl_ = −ΔE_c_/e = 0. ΔV_sem_ = ΔV_appl_, indicating that C_q_ ≪ C_edl_ (that is, the E_F_ is located in the band gap with zero DOS near the E_F_). In this case, E_c_ is pinned at a fixed value in the energy coordinates, and E_F_ moves to the band edge of E_c_ at the maximum rate (Fig. 2C, iv, iii and ⅱ). However, the transition from type I to type II potential drop occurs at an onset potential of approximately −0.15 V, indicating that the E_F_ is tuned into the bottom of the E_c_ at V_appl_ = −0.15 V (Fig. 2C, ⅱ). This results in an increase in C_q_ in the basal plane of ML MoS_2_ due to the large DOS near E_F_, as shown in the top panel of Fig. 2B. When V_appl_ < −0.15 V, ΔV_appl_ = −ΔE_F_/e, ΔV_edl_ = −ΔE_c_/e and ΔV_sem_ = ΔV_appl_−ΔV_edl_. The E_c_ starts to move. As V_appl_ becomes more negative, E_c_ moves faster. In this situation, the relative rate between E_F_ and E_c_ gradually decreases, as shown in Fig. 2C, i. However, the difference in the movement rate between E_F_ and E_c_ still exists, which means that the Fermi level keeps moving into the conduction band. These results are inconsistent with the classical theory that the Schottky-analogue junction is broken once the E_F_ is tuned into the conduction band, and it cannot be further tuned deep in bands [32]. The bandgap E_gap_ = 1.85 eV of ML MoS_2_ can be determined from photoluminescence spectroscopy (PL), depicted in Fig. S10C. Since the energy difference between E_F_ and E_c_ determines the surface charge concentration (n_s_), for 2D semiconductor materials, n_s_ can be calculated by (see Supplementary Data for derivation details):


(1) \begin{eqnarray*} {N_S} \cdot = {N_{\rm{C}}} \cdot {\rm ln}\left[ {1 + {\rm exp}\left( { - \frac{{{E_{\rm{C}}} - {E_{\rm{F}}}}}{{{k_{\rm{B}}}T}}} \right)} \right], \end{eqnarray*}

where N_c_ ∼8.6 × 10^12^ cm^−2^ is the effective density of states in the conduction band [40]. The conductivity of the basal plane is constantly changing since the relative positions of E_F_ and E_c_ in the energy coordinates change with the V_appl_. Figure 2D plots the relationship between n_s_ and V_appl_. The n_s_-V_appl_ curve shows the switching effect of n_s_: the surface is turned ‘on’ with high electron concentration (over 1 × 10^13^ e cm^−2^ in the basal plane of ML MoS_2_, consistent with the ‘liquid gating’ effect [13,35,41,42]) at V_appl_ below −0.15 V, and is turned ‘off’ with low electron concentration (insulating) V_appl_ higher than −0.15 V. The results show that the surface electron concentration can be effectively tuned under electrolyte conditions. Through the *in-situ* surface potential measurement, we visualized the evolution of the band structure under electrode conditions and how the high conductivity on the semiconductor surface occurs.

Moreover, it can be seen from the n_s_-V_appl_ curve that the basal plane of ML MoS_2_ is nearly insulated at the open circuit potential (∼0.14 V). It corresponds to the depleted state of ML MoS_2_ with low electron concentration in solution (the flat band potential is approximately equal to −0.07 V, estimated from a macroscopical Mott-Schottky measurement, Fig. S9).

The switching effect of n_s_ in the basal plane was further proven by conductivity measurements [42–44]. An *in-situ* local electric conductivity measurement was performed, shown in the inset of Fig. 2D. With the tip directly contacting the surface of the basal plane (lift = 0, substrate generation/tip collection (SG/TC) mode), a linear sweep voltammetry (LSV) curve was collected on the substrate while the potential of the tip remained constant. In this way, the conducting current *I* is recorded at the solid–liquid interface (containing only the supporting electrolytes to exclude the Faradic reaction). It is shown that the basal plane of ML MoS_2_ becomes conductive (‘on’) once the V_appl_ is below the onset potential of approximately −0.15 V. Otherwise, the basal plane maintains an insulating state (‘off’). In previous studies, the onset potential of the conductivity of low DOS semiconductor materials (2D WS_2_, MoS_2_, etc.) was regarded as the potential at which E_F_ reaches the position of the band edge of E_c_ [13,45], proving the accuracy of the surface potential measurement. To date, how the band structure of the semiconductor is transformed under electrolyte conditions has not been clearly studied by conventional electrochemical methods. The difficulty is in distinguishing the potential distribution between semiconductor and EDL, which still depends on theoretical calculations. In our study, the potential drops in the semiconductor (ΔV_sem_) and the EDL (ΔV_edl_) are decoupled by direct measurements. Based on the potential values, the evolution of band structure can be revealed under electrolyte conditions.

### The role of the applied voltage in electrocatalytic reactions

Electron transfer (ET) and the subsequent formation and rupture of chemical bonds (catalytic reaction) are two fundamental processes in electrocatalytic reactions [9]. However, it is difficult to distinguish their contributions from each other due to their convoluted nature. It becomes even more challenging for non-metal electrocatalysts with spatial heterogeneity in electronic structures and catalytic centers [46,47].

**Figure 3.**
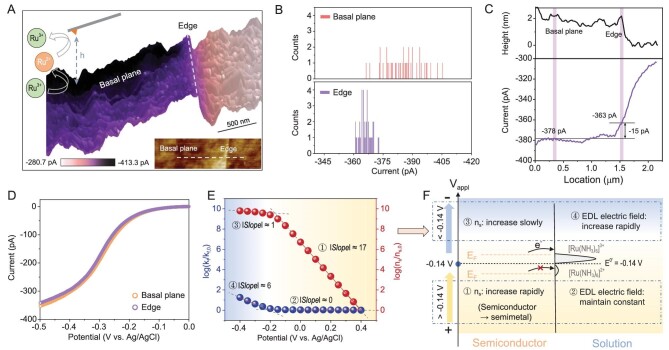
ET imaging on the ML MoS_2_ electrocatalyst. SG/TC mode was used in this experiment. The Pt tip and substrate were used as dual-working electrodes. Semi-ring graphite and Ag/AgCl (saturated KCl) were used as the counter electrode and reference electrode, respectively. Products ([Ru(NH_3_)_6_]^2+^) are generated from the negatively biased substrate and oxidized to reactants ([Ru(NH_3_)_6_]^3+^) by a positively biased tip. (A) ET images of ML MoS_2_. ET currents are superimposed on a 3D topography map. Inset, 2D topography map of ML MoS_2_. Fixed height h = 25 nm, E_Sub_ = −0.5 V, E_Tip_ = 0.3 V (vs. Ag/AgCl). The solution contains 5 mM [Ru(NH_3_)_6_]Cl_3_ and 0.1 M KCl. P^++^-Si was used as substrate. (B) Histograms of ET current distributions at different sites. (C) Values of topography and ET current obtained along the dashed line in the inset of (A). (D) Spatially resolved LSV curves obtained on the edge and basal plane of ML MoS_2_ for the [Ru(NH_3_)_6_]^3+^/[Ru(NH_3_)_6_]^2+^ system (h = 25 nm, E_Tip_ = 0.3 V (vs. Ag/AgCl), v = 3 mV/s). We set the cathode current to be positive and the anodic current to be negative. (E) The potential-dependent behavior of the rate constant k_f_ and the surface electron concentration n_s_ in the ET reaction are further decoupled based on the measurements of ΔV_sem_ and ΔV_edl_. (F) Schematic of the potential-dependent behavior of k_f_ and n_s_ in (E).

To study how V_appl_ acts in an electrocatalytic reaction, first we used outer-sphere redox pairs to decouple the ET process from the electrocatalysis process and figure out how the V_appl_ drives the ET process. Atomic-force-microscope-based scanning electrochemical microscopy (AFM-SECM) [24,48,49] (Fig. S11) was used to map the local electrochemical activity of ML MoS_2_ electrocatalysts in SG/TC mode [50,51]. A higher *i_Tip_* reflects a higher local product concentration (that is, higher local electrochemical activity) of the electrocatalysts. The outer-sphere redox pairs with the reaction [Ru(NH_3_)_6_]^3+^ + e^−^ → [Ru(NH_3_)_6_]^2+^ were used to provide information about the ET [20]. The ET image clearly shows that the basal plane of MoS_2_ has comparable ET activity to that of the edge sites (Fig. 3A). The ET currents in the back of the topographic image are a bit higher than those in the front, due to the change in local concentration of redox pairs near the surface during the imaging process (scanning from back to front in Fig. 3A). Histograms of ET current distributions at different sites are shown in Fig. 3B. The ET currents taken from the single scan line collected in a short time are comparable. Figure 3C gives the topography and ET current values obtained along the same scan line (the dashed line in Fig. 3A). The current values of the basal plane and the edge differ by only 15 pA. The spatially resolved LSV curve of the basal plane largely matches that of the edge (Fig. 3D), indicating no significant difference in the ET rate between basal plane and edges at different biases. ET imaging shows high ET activity of the basal plane, contrary to the traditional consensus under *ex-situ* conditions [52,53]. It is reasonable to speculate that the high ET activity may derive from the aforementioned ‘liquid gating’ effect with high n_s_. However, ET currents are jointly decided by both the n_s_ and rate constant k_f_ (*i* ∝ n_s_ and k_f_) [1], that is


(2) \begin{eqnarray*}i = {\rm{F}}\cdot{\rm{A}}\cdot{{\rm{n}}_{\rm{s}}}\cdot{{\rm{k}}_{\rm{f}}}\cdot{{\rm{C}}_0},\end{eqnarray*}

where F is Faraday constant, A is electrode area, n_s_ is surface electron concentration related to surface electrical conductivity, and C_0_ is the reactant ([Ru(NH_3_)_6_]Cl_3_) concentration near the surface of the electrode. Here, the reverse reaction is negligible. The rate constant k_f_ can be expressed as k_0_·exp[−αf(E_s_–E^0'^)], in which k_0_ is the standard rate constant, E_s_ is surface potentials of the electrode, α is the transfer coefficient, ${\rm{f}} = \frac{{\rm{F}}}{{{\rm{RT}}}}$, and E^0'^ is the formal potential. In a solution containing 5 mM [Ru(NH_3_)_6_]^3+^, the formal potential E^0′^, estimated as E_1/2_ of the metal substrate, is −0.14 V for [Ru(NH_3_)_6_]^3+/2+^ (Fig. S12B), which is close to the V_appl_ at which MoS_2_ becomes semi-metallic. Only when the voltage is applied to change the n_s_, rather than k_f_ for the basal plane, can the ‘liquid gating’ effect be responsible for the high ET activity. To date, it is still unclear how much the n_s_ contributes to the ET rate in non-metal electrocatalysts.

Herein, the electronic structure of the basal plane measured in supporting electrolytes (without Faraday currents) was used to predict the factors affecting the ET activity and give a meaningful reference. According to k_f_ = k_0_·exp[−αf(E_s_–E^0′^)], since k_0_ is unknown it is hard to obtain the specific value of k_f_. However, k_f_ at different V_appl_ can be compared by k_f_(E_s,1_)/k_f_(E_s,2_) = exp[−αf(E_s,1_–E_s,2_)]. Taking k_f_ at V_appl_ = 0.4 V as the reference point (marked as k_f,0_), we can obtain k_f_/k_f,0_ at different V_appl_. Similarly, we can also have n_s_/n_s,0_ at different V_appl_ (the reference point n_s,0_ is n_s_ at V_appl_ = 0.4 V). Combining k_f_/k_f,0_ and n_s_/n_s,0_, the potential-dependent behavior of k_f_ and n_s_ can be obtained (Fig. 3E). As shown in Fig. 3F, when V_appl_ is more positive than the formal potential E^0′^ (V_appl_ > −0.14 V), the electrochemical potential (or E_F_) of MoS_2_ is lower than that of the [Ru(NH_3_)_6_]^3+/2+^ redox pair and there is no driving force to transfer electrons from MoS_2_ to the solution (ET reaction does not occur). In this voltage range, all potential drops within the semiconductor increase the n_s_ rapidly with a |Slope| ≈ 17 for log(n_s_/n_s,0_). There are no potential drops within the EDL, so the electric field intensity of EDL remains constant and k_f_ does not change with |Slope| ≈ 0 for log(k_f_/k_f,0_). In this voltage range, the effect of the applied voltage can be considered as a ‘preparation’ for the ET reaction, transforming MoS_2_ from a semiconductor state to a semi-metallic state. When V_appl_ is more negative than the formal potential E^0′^ (V_appl_ < −0.14 V), the electrochemical potential of MoS_2_ is higher than the [Ru(NH_3_)_6_]^3+/2+^ redox pair, creating a driving force for electrons to transfer from MoS_2_ to solution, and the ET reaction begins to occur. In this voltage range, the potential drop within the EDL gradually increases. The V_appl_ begins to act on the electric field intensity of EDL, changing the value of k_f_. When V_appl_ < −0.3 V, the V_appl_ increases k_f_ rapidly with |Slope| ≈ 6 for log(k_f_/k_f,0_), while the V_appl_ increases n_s_ slowly with |Slope| ≈ 1 for log(n_s_/n_s,0_). When the ET reaction occurs, the effect of the applied voltage is to a greater extent to change the k_f_ value, prompting electrons to pass through the solid–liquid interface for the ET reaction.

**Figure 4.**
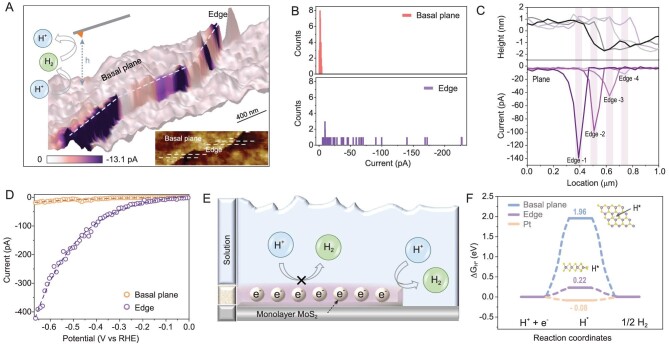
HER imaging on the ML MoS_2_ electrocatalyst. Products (H_2_) are generated from the negatively biased substrate and oxidized to reactants (H^+^) by a positively biased tip. (A) HER images of ML MoS_2_. HER currents are superimposed on a 3D topography map. Inset, 2D topography map of ML MoS_2_. Fixed height h = 25 nm, E_Sub_ = −0.5 V, E_Tip_ = 0.3 V (vs. Ag/AgCl). The solution contains 10 mM HClO_4_ and 0.1 M NaClO_4_. p^++^-Si were used as substrate. (B) Histograms of HER current distributions at different sites. (C) Values of topography and HER current obtained along the dashed lines in the inset of (A). (D) Spatially resolved LSV curves obtained on the edges and basal plane of ML MoS_2_ for the H^+^/H_2_ system. The tip was biased at 0.3 V (vs. Ag/AgCl) at a fixed height, h = 25 nm, while LSV was performed on the substrate (v = 5 mV/s). We set the cathode current to be positive and the anodic current to be negative. The dashed line in (D) is the fitting results according to the respective current values. (E) A model of ML MoS_2_ in the HER. (F) Comparison of the Gibbs free energies of the adsorbed H on the basal plane and edge of MoS_2_.

The performance of the ML MoS_2_ in an electrocatalytic (inner-sphere) reaction was also investigated at the nanoscale. Figure 4A displays HER current mapping superimposed on a 3D topography map. The result shows that the high tip current (*i_Tip_*) is collected at the edge sites, while the current at the basal plane is weak. Histograms of HER current distribution at different sites are shown in Fig. 4B. Compared with the outer-sphere reaction, the inner-sphere reaction is more sensitive to the chemical properties of the active site. The HER currents at different edge sites vary greatly, ranging from a few pA to hundreds of pA. The differences in HER currents reflect the variation in the chemical properties of edge sites under *in-situ* conditions. Figure 4C gives four current-topography plots along the dashed lines in Fig. 4A, representing the edge sites with different HER activities. Despite differences in the current, the topography of the edge corresponds exactly to the maximum of the HER current. In addition, the spatially resolved LSV method was also performed for ML MoS_2_ (Fig. 4D). The basal plane is almost catalytically inert over a wide potential range, while the electrochemical currents at the edge increase exponentially with increasing negative bias. In a previous study, Norskov *et al.* found that the HER activity of MoS_2_ is proportional to its edge length, and thus hypothesized that the edges are the active sites of the HER [15]. Our work provides direct evidence for the structure–activity relationship of MoS_2_ through *in-situ* HER imaging. With the outer-sphere and the inner-sphere reaction, the spatial mismatch of charge transfer and chemical reaction processes of ML MoS_2_ was clearly revealed at the nanoscale. The applied voltage plays an essential role in the electron transfer step, increasing the surface electron concentration n_s_ and the rate constant k_f_ of the basal plane. However, the large number of electrons reaching the surface cannot participate in the chemical reactions (Fig. 4E). The large Gibbs free energy of the atomic hydrogen (ΔG_H*_) adsorbed on the basal plane (1.96 eV) prevents chemical processes at the solid–electrolyte interface (Fig. 4F). Therefore, the low binding energy of H contributes to low HER rates through the effect of high barriers, resulting in low energy-conversion efficiency.

## CONCLUSIONS

Through *in-situ* surface potential measurements, the potential distribution between the basal plane of ML MoS_2_ (ΔV_ch_) and electrolyte (ΔV_edl_) was decoupled quantitatively as a function of the applied voltage. We visualized the evolution of the band structure under electrode conditions and revealed the process of conductivity transformation on the semiconductor surface. To clarify how the applied voltage acts on the electrocatalytic reaction, the ET and catalytic reaction processes on ML MoS_2_ were identified at the nanoscale through AFM-SECM mapping. We clearly showed that the applied voltage plays an essential role in the ET process of the basal plane of MoS_2_, increasing the surface electron concentration n_s_ and the rate constant k_f_ to different degrees under different V_appl_. However, the applied voltage cannot be precisely applied to the electrocatalytic reaction due to the spatial mismatch of charge transfer and reactant adsorption sites. This work paves the way for the rational design of efficient non-metallic electrocatalysts based on the understanding of how voltage acts on non-metallic catalysts at the nanoscale.

## Supplementary Material

nwad166_Supplemental_File
