# Supplementary material for: Visualizing the role of applied voltage in non-metal electrocatalysts
Source: Natl Sci Rev. 2023 Jun 6;10(9):nwad166. doi: 10.1093/nsr/nwad166 (PMC10411668; doi:10.1093/nsr/nwad166)
Supplement: nwad166_Supplemental_File [file nwad166_supplemental_file.pdf]

Supplementary Materials

Visualizing the Role of Applied Voltage in Non-metal Electrocatalyst

Ziyuan Wang[1,2], Jun Chen[1,3], Chenwei Ni[1,3], Wei Nie[1,3], Dongfeng Li[1,3], Na Ta[1], Deyun Zhang[1,2], Yimeng Sun[1,2], Fusai Sun[1,2], Qian Li[1,2], Yuran Li[1,2], Ruotian Chen[1], Tiankai Bu[4], Fengtao Fan[1,*] and Can Li[1,*]

[1]Collaborative Innovation Center of Chemistry for Energy Materials (iChEM), State Key Laboratory of Catalysis, Dalian National Laboratory for Clean Energy, Dalian Institute of Chemical Physics, Chinese Academy of Sciences, Dalian 116023, China.

[2]Collaborative Innovation Center of Chemistry for Energy Materials (iChEM), College of Chemistry and Chemical Engineering, Xiamen University, Xiamen 361005, China.

[3]University of Chinese Academy of Sciences, Beijing 100049, China

[4]Department of Materials, Imperial College London, SW7 2AZ, United Kingdom.

*__Corresponding authors__. E-mails: ftfan@dicp.ac.cn; canli@dicp.ac.cn

This PDF file includes:

Materials and Methods
Figs. S1 to S15
References

Materials and Methods

1. Synthesis of monolayer (ML) MoS_2

The ML MoS_2 crystals grown on a sapphire substrate were obtained commercially from SixCarbon Technology, Shenzhen and produced by CVD method:

MoO_3 (Alfa Aesar) was used as the molybdenum source, sulfur powder (Alfa Aesar) was used as the sulfur source, Ar was used as the growth carrier gas, and the sapphire substrate was placed face down on a crucible containing MoO_3 powder. A double-temperature tube furnace with a diameter 80 mm diameter was used. During the reaction, MoO_3 was heated to 650 ℃, sulfur was heated to 180 ℃, and triangular MoS_2 crystals were grown on the sapphire substrate. As the MoS_2 growth time increases, the triangular MoS_2 crystals were interconnected to form semi-continuous film or continuous films.

2. Electrode preparation

To eliminate the strain introduced by epitaxial growth on sapphire substrates and to study the intrinsic properties of ML MoS_2, the ML MoS_2 film was peeled off from the sapphire substrate and then transferred to a conductive substrate (Au (Si-10 nm Ti-50 nm Au) or Si (P type; 0.001-0.005 ohm·cm) using deionized water (18.2 MΩ cm) or PMMA method.

3. Deionized water transfer method

The ML MoS_2 film was peeled off directly from the sapphire substrate using deionized water and then transferred to a conductive substrate. Finally, the sample was heated at 100 ℃ for 10 minutes. This method can avoid the introduction of organic impurities and maintains the intrinsic properties of MoS_2. The MoS_2 transferred by this method was used for *in-situ* tests at nanoscale.

4. PMMA transfer method

The PMMA anisole solution was dripped onto ML MoS_2 film and heated at 100 ℃ for 10 min. Afterwards, the PMMA-coated sample was soaked in 2 M NaOH solution for 2 hours. PMMA-MoS_2 was gradually peeled off from the sapphire substrate. After cleaning with deionized water, PMMA-MoS_2 was placed on a conductive substrate and heated at 80 ℃ for 10 min. To remove PMMA, the MoS_2 sample was soaked in acetone for about 30 minutes each time by repeating

this procedure three times. Finally, the sample was heated at 100 ℃ for 10 minutes. The MoS_2 transferred by this method was used for Mott-Schottky and Capacitance-Voltage tests.

5. Home-built *in-situ* surface potential characterization technique

Electrochemical tests were carried out by a CHI760E electrochemical workstation. A three-electrode system was used for electrochemical reaction with Ag/AgCl (saturated KCl) as reference electrode, graphite as counter electrode and MoS_2 electrode as working electrode. The Pt/Ir probe was manipulated through an AFM positioning system to contact the surface of the working electrode and measure the local surface potential V_s. The local surface potential V_s relative to an Ag/AgCl reference electrode under different applied voltages was recorded by a high impedance (1 TΩ) amplifier with negligible leakage current. The measured surface potential difference was assigned to inner potential difference at solid/liquid interfaces[1]. In this case, the change in potential drop across the EDL (ΔV_{edl}) can be read directly from $V_s(V_{appl} \neq 0)$ - $V_s(V_{appl} = 0)$. By combining with the change of back electrode potential (ΔV_{appl}), the change in potential drop across electrocatalyst (ΔV_{sem}) can also be obtained from $\Delta V_{sem} = \Delta V_{appl} - \Delta V_{edl}$. To eliminate the effect of redox reactions on the tip, we used a solution containing only supporting electrolytes and studied the intrinsic properties of ML MoS_2 at different applied voltages.

6. *In-situ* electrochemical imaging setup

The *in-situ* electrochemical imaging system was employed an atomic force microscope (AFM; Bruker Dimension Icon) combined with a scanning electrochemical microscope (SECM) module, which allows simultaneous acquisition of topography and current signals to provide accurate structure-activity information. Commercial PeakForce SECM (Pt) probes and PeakForce tapping mode were used in liquid phase. An electrochemical workstation (CHI 760E) was used to control the voltage and transmit current signals to the AFM system for electrochemical imaging.

To identify the ET and HER sites respectively, the outer-sphere redox pairs ($[Ru(NH_3)_6]^{3+}/[Ru(NH_3)_6]^{2+}$) and the inner-sphere ($H^+/H_2$) reaction were used. Here, substrate generation/tip collection (SG/TC) mode was adopted to reflect the real catalytic reaction process. The tip and substrate were used as dual-working electrodes and graphite and Ag/AgCl (saturated KCl) were used as counter electrode and reference electrode respectively. Electroactive species

are generated at the negatively biased substrate ($O + e^- \rightarrow R$) and collected at a positively biased tip ($R - e^- \rightarrow O$).

Outer-sphere ($[Ru(NH_3)_6]^{3+}/[Ru(NH_3)_6]^{2+}$) electrochemical reaction imaging

MoS_2: $[Ru(NH_3)_6]^{3+} + e^- \rightarrow [Ru(NH_3)_6]^{2+}$ (Cathodic reaction)

SECM tip: $[Ru(NH_3)_6]^{2+} \rightarrow [Ru(NH_3)_6]^{3+} + e^-$ (Anodic reaction)

Since the Outer-sphere redox pairs do not bond to the surface of electrode, $[Ru(NH_3)_6]^{3+/2+}$ redox pairs can be used to study the heterogeneous electron transfer (ET) sites distribution.

Anodic currents through the tip (i_{Tip}) were detected (in this study, cathode current was set to be positive and anodic current was set to be negative). A higher i_{Tip} reflects a higher local product concentration (higher local electrochemical activity) of the electrocatalysts.

Inner-sphere (H^+/H_2) electrochemical reaction imaging

MoS_2: $2H^+ + 2e^- \rightarrow H_2$ (Cathodic reaction)

SECM tip: $H_2 \rightarrow H_2 + 2e^-$ (Anodic reaction)

In acidic solution, the Volmer step ($H^+ + e^- + * \rightarrow H^*_{ads}$) is a necessary process to generate adsorbed hydrogen atoms (H^*_{ads}), regardless of whether the hydrogen evolution reaction (HER) is carried out by the Volmer-Tafel mechanism or the Volmer-Heyrovsky mechanism. It means that HER is an Inner-Sphere reaction that reacts at the Inner Helmholtz Plane (IHP). The active sites where HER occurs are both ET sites and H-adsorption sites. The distribution of chemical sites (H-adsorption) can be obtained in comparison with the distribution of ET sites of MoS_2.

During the imaging process, surface topography was recorded by the main scan, followed by a lift scan with a fixed tip-substrate distance to obtain the electrochemical currents, eliminating the convolution of morphology to electrochemical currents.

7. *In-situ* electric conductivity measurement

PeakForce SECM probes and contact mode were used for *in-situ* electric conductivity measurement. In contrast to the imaging mode where the probe was lifted to a certain height to collect the products, in the electric conductivity measurement mode, the probe is maintained on the catalyst surface without being lifted. In this manner, the conductivity current *I* is recorded at the solid-liquid interface (containing only supporting electrolytes to exclude the Faradic reaction).

With the tip directly contacting the basal plane of ML MoS_2 (lift=0, SG/TC mode), LSV was collected on the substrate while the potential of the tip was constant).

8. DFT Computational

First-principles calculations based on Density Functional Theory (DFT) were carried out by using the Vienna Ab initio Simulation Package (VASP)[2,3]. The interactions between valence electrons and ions were treated with the projector-augmented wave (PAW) method[4]. The exchange-correlation interactions were described by generalized gradient approximation (GGA)[5] with the Perdew-Burke-Ernzerhof (PBE) functional[6]. The electron wave functions were expanded in a plane-wave basis set with cutoff energy of 520 eV. The convergence criterion for residual force on each atom during structure relaxation was set to 0.02 eV/Å. And the geometries were relaxed to minimize the total energy of the system until a precision of 10^{-5} eV was reached.

According with previous studies of 2H MoS_2, 50% S coverage of Mo-edge is the most stable form under HER conditions[7]. We created p (4 × 4) MoS_2 single layer, and 4-layer slab models of $(10\bar{1}0)$ 50% S coverage of Mo-edge and $(\bar{1}010)$ S-edge with at least 15 Å vacuum spaces from a fully optimized single-layer hexagonal MoS_2. A 13 × 13 × 1 Monkhorst-Pack k-mesh grid was used for k-points sampling of the unit cell in the single-layer calculations, while 4 × 4 × 1, 4 × 1 × 1 and 1 × 4 × 1 Monkhorst-Pack k-mesh grids were used in the optimization of the basal plane, the Mo-edge slabs and S-edge slabs, respectively. During the geometry optimization and energy calculations of Mo-edge and S-edge slabs, the bottom two layers of the slabs were fixed while the top two layers of the slabs, including the adsorbates, were fully relaxed.

The methodology of the calculation for Pt is similar. The Pt(111) surface was modelled with a 2 × 2 unit cell. 4 × 4 × 1 Monkhorst-Pack mesh k-mesh grids were used. Pt(111) were modelled as a four-layer slab with the bottom two layers fixed during optimization.

The hydrogen adsorption free energy changes were derived using the principles of material and energy balance:

$$\Delta G_{H*} = \Delta E_H + \Delta E_{ZPE} - T\Delta S_H$$

Where ΔE_H is the H adsorption energy calculated by the following equation.

$$\Delta E_H = 1/2\ [E(surf+*H)-E(surf)-1/2 \cdot E(H_2)]$$

Where n is the number of the adsorbed H atoms in the supercell, E(surf+*H) and E(surf) are the energies of the supercells with and without adsorbed H. $E(H_2)$ is the energy of a free H_2 molecule.

ΔE_{ZPE} is the difference in zero-point energy between the adsorbed H and the gas phase. The vibrational entropy in the adsorbed state is small and the entropy difference $\Delta S_H \cong -1/2\ S_{H2}^0$, where S_{H2}^0 is the entropy of H_2 in the gas phase at standard conditions as discussed in previous literature[8], corresponding to 1 bar of H_2 at room temperature. The contribution of the catalyst to ΔE_{ZPE} and ΔS_H are small and can be neglected. Our calculations give ΔE_{ZPE} to be 0.096 eV and $T\Delta S_H$ to be -0.197 eV at room temperature, so we have $\Delta G_{H^*} = \Delta E_H + 0.283$ eV.

9. Characterization

The topography of the SECM probe was observed by scanning electron microscopy (SEM; Quanta 200 FEG). Annular dark-field (ADF) scanning transmission electron microscopy (STEM) was performed using JEOL Grand ARM operating at 80 kV. The chemical composition of ML MoS_2 was analyzed using X-ray photoelectron spectroscopy (XPS; Thermofisher ESCALAB 250Xi instrument) with an Al Kα excitation source. The Raman and PL measurements were performed with the excitation laser line of 532 nm using a Metatest Scanpro laser scanning microscope. Mott-Schottky plot and Capacitance-voltage curve were carried out by a VersaSTAT 4 electrochemical workstation (1k Hz; 0.1 M K_2SO_4).

10. The surface electron concentration (n_s) for two-dimensional semiconductor materials

Considering the band structure as:

$$E = \frac{\hbar^2 k^2}{2m^*} \#(1)$$

Where m^* is the effective mass. The free electron gas states number can be written as:

$$N(\epsilon) = \frac{2}{(2\pi)^2} \cdot \pi \frac{2m^*\epsilon}{\hbar^2} \cdot g_v = g_v \cdot \frac{m^*}{\pi\hbar^2} \cdot \epsilon \#(2)$$

Where g_v is the valley degeneracy. The density of states:

$$g(\epsilon) = g_v \cdot \frac{m^*}{\pi\hbar^2} \#(3)$$

As fermions, the electron distribution in 2D electron gas conforms to the Fermi-Dirac distribution, so the conduction band electron concentration can be written as:

$$n = \int_0^\infty g_v \cdot \frac{m^*}{\pi\hbar^2} \cdot \frac{1}{1 + \exp\left(\frac{\varepsilon - \zeta}{k_B T}\right)} \cdot \mathrm{d}\varepsilon \ \#(4)$$

Where $\varepsilon = \epsilon - E_{\mathrm{C}}$, $\zeta = E_{\mathrm{F}} - E_{\mathrm{C}}$, so we can get:

$$n = g_v \frac{m^* k_{\mathrm{B}} T}{\pi \hbar^2} \ln\left[1 + \exp\left(-\frac{E_{\mathrm{C}} - E_{\mathrm{F}}}{k_{\mathrm{B}} T}\right)\right] = N_{\mathrm{C}} \cdot \ln\left[1 + \exp\left(-\frac{E_{\mathrm{C}} - E_{\mathrm{F}}}{k_{\mathrm{B}} T}\right)\right] \#(5)$$

Where $N_{\mathrm{C}} = g_v \frac{m^* k_{\mathrm{B}} T}{\pi \hbar^2}$ is the 2D effective density of states in the conduction band.

Since a 2D semiconductor is thin enough that the width of the space charge region can be ignored, its electron concentration (n) can be considered to be the surface electron concentration (n_s). Equations (5) can be applied to both the nondegenerate and the degenerate semiconductors.

When $E_{\mathrm{C}} \gg E_{\mathrm{F}}$, from (5) we can get:

$$n = N_{\mathrm{C}} \cdot \exp\left(-\frac{E_{\mathrm{C}} - E_{\mathrm{F}}}{k_{\mathrm{B}} T}\right) \#(6)$$

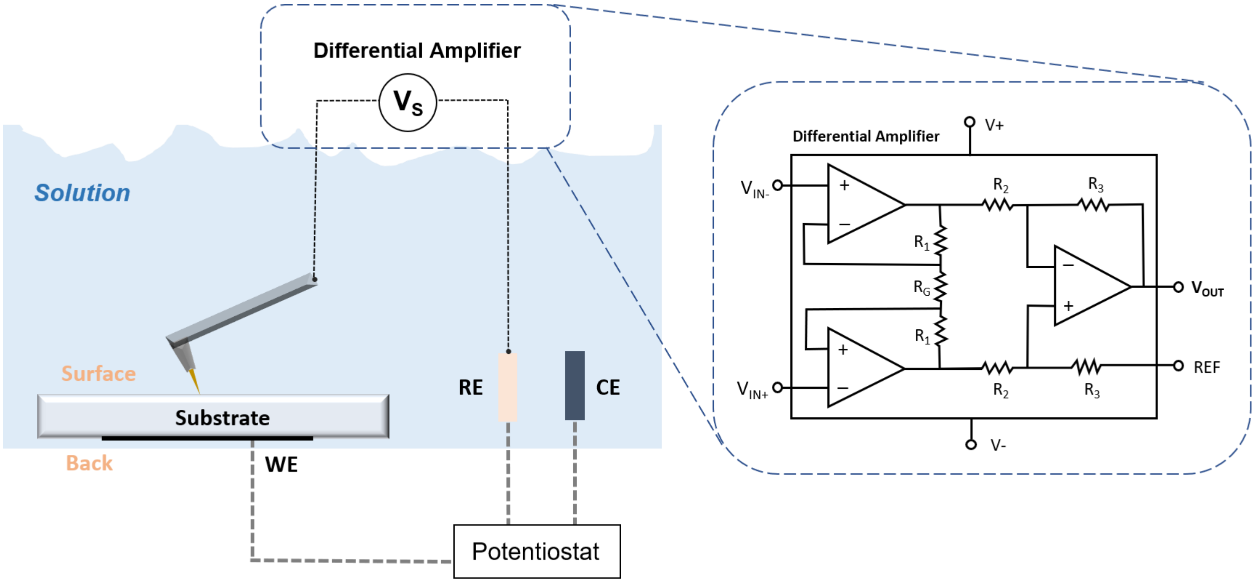

Fig. S1. Schematic of a home-built *in-situ* surface potential measurement setup and the equivalent electronic circuitry of differential amplifier.

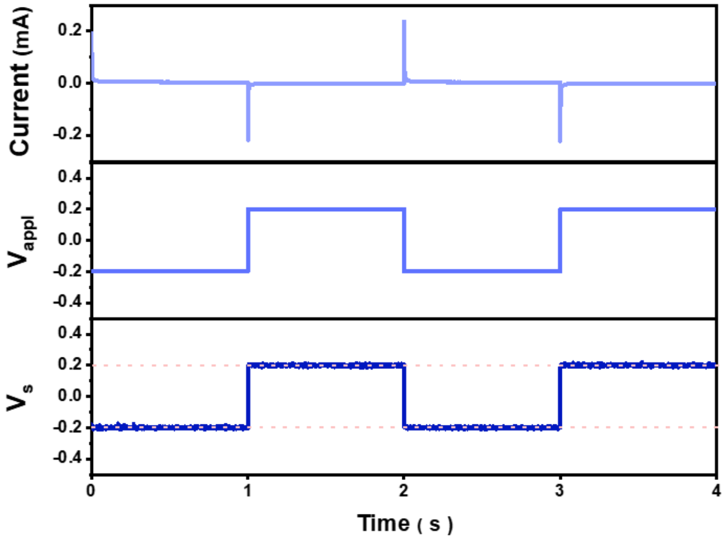

Fig. S2. *In-situ* surface potential measurement on Au substrates. The local surface potential V_s (vs. Ag/AgCl) of Au recorded by a conductive tip tracked the applied potential in real-time response. Cathode current was set to be positive and anodic current was set to be negative.

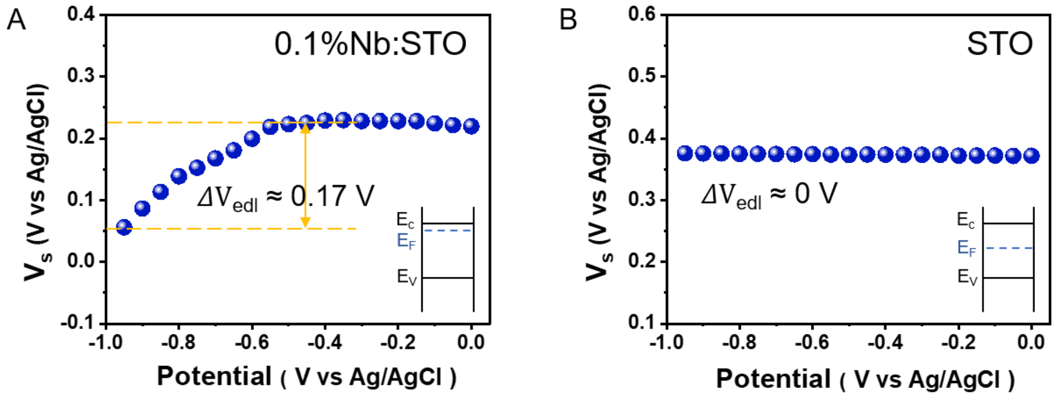

Fig. S3. *In-situ* surface potential measurement on 0.1%Nb:STO and STO crystals. (A) For N-type STO crystal (the Fermi level E_F is closer to the bottom of the conduction band E_c), the surface potential changes as the applied voltage becomes negative. (B) For intrinsic STO crystal (the Fermi level E_F lies in the middle of the band gap E_g), the surface potential doesn't change at negative voltage.

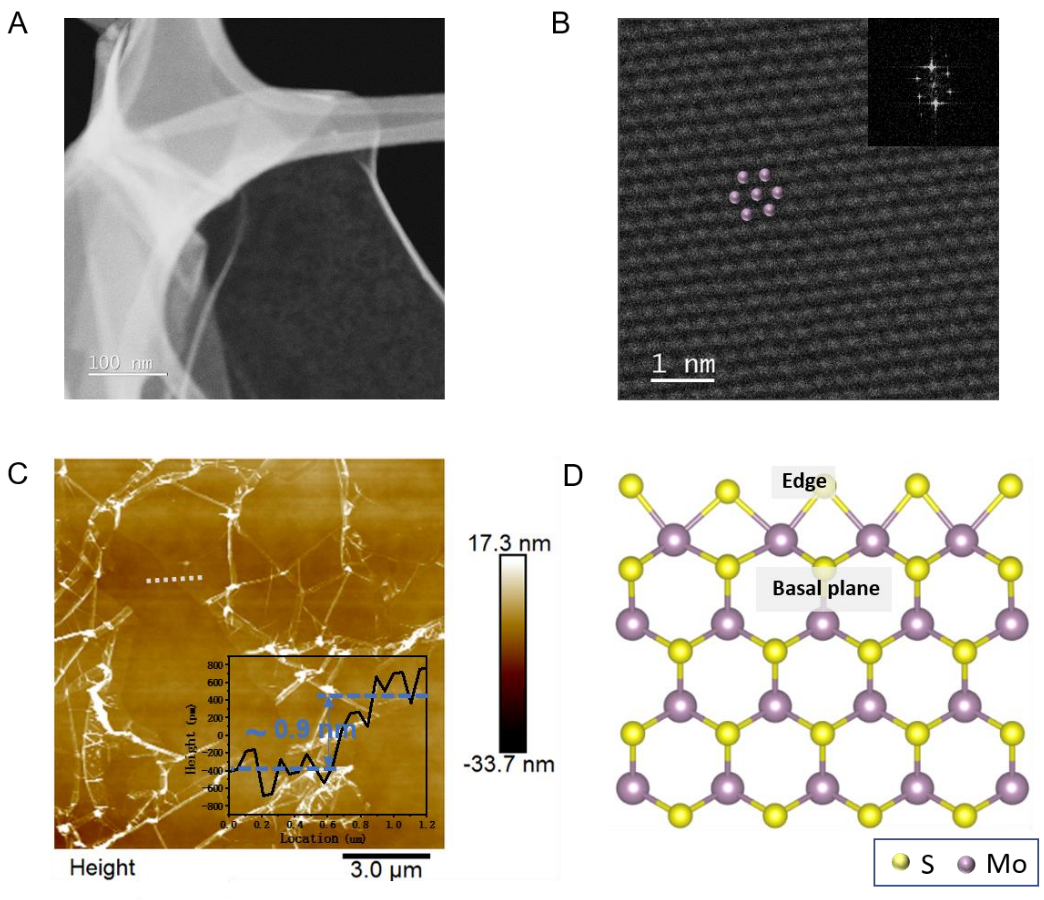

Fig. S4. The topography of ML MoS_2. (A-B) STEM ADF imaging of ML MoS_2 film. The position of the brightest spots reflects the atomic positions of Sulphur (S) atoms. Inset of (B), Fourier transform image of the lattice. (C) The AFM image of ML MoS_2 film transferred on Si substrate by deionized water transfer method. Inset of (C), Height profiles of the flake marked in the figure. (D) The atomic model of ML MoS_2.

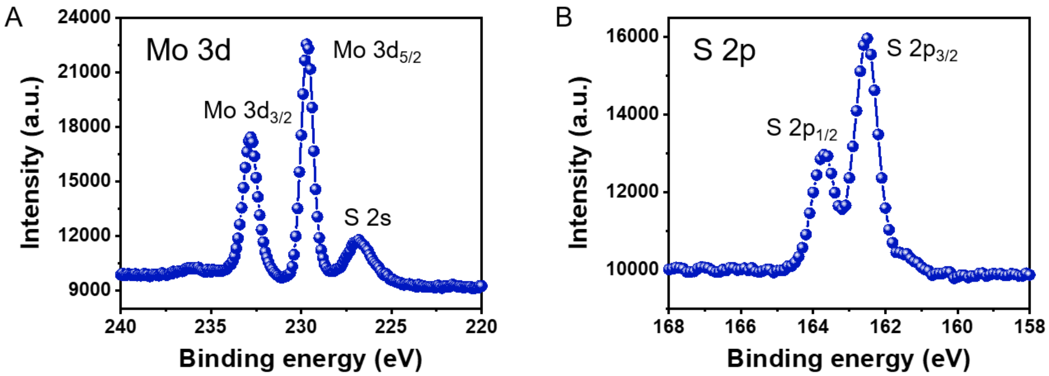

Fig. S5. XPS data of ML MoS_2.

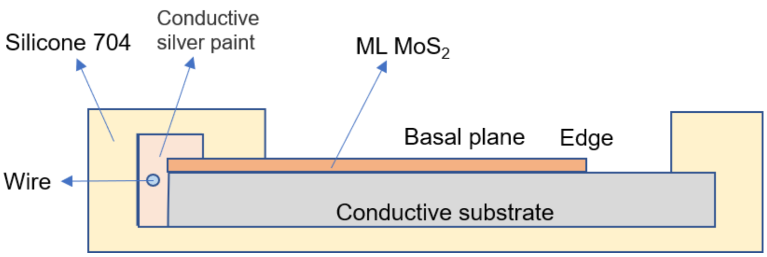

Fig. S6. Schematic diagram of ML MoS_2 electrode. ML MoS_2 film was transferred to the conductive substrate. Conductive silver paint was used to connect the wire to one side of the electrode. Finally, most of the electrode area was covered with silicone 704 to isolate the solution.

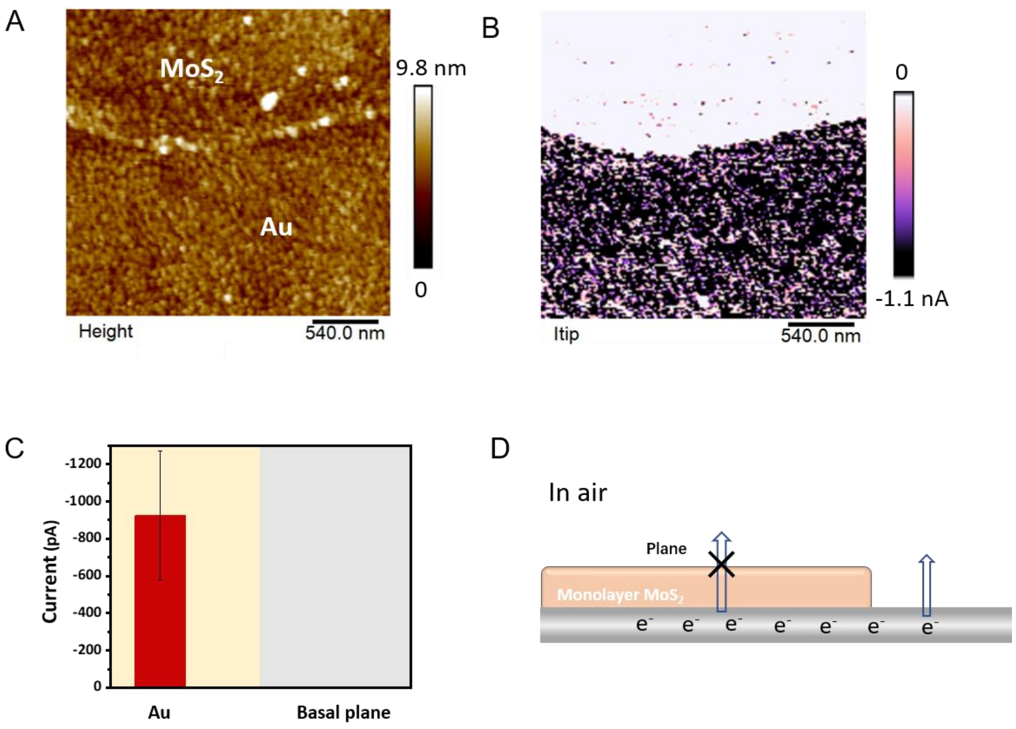

Fig. S7. Conductivity map of ML MoS_2 in air conditions. 2D topography map (A) and conductivity map (B) of ML MoS_2 were captured in air by bipotentiostat (CHI 760E) using a Pt/Ir tip. The substrate was used as WE and the tip was used as CE and RE. To protect the tip from high currents, a 10 MΩ resistor is connected to the circuit. A bias voltage (V= -0.2 V vs. tip) was applied to the substrate and the current was collected by the Pt/Ir tip. Contact mode was used. (C) Conductive current histogram of Au and the basal plane of ML MoS_2. (D) Schematic of conductive model of ML MoS_2.

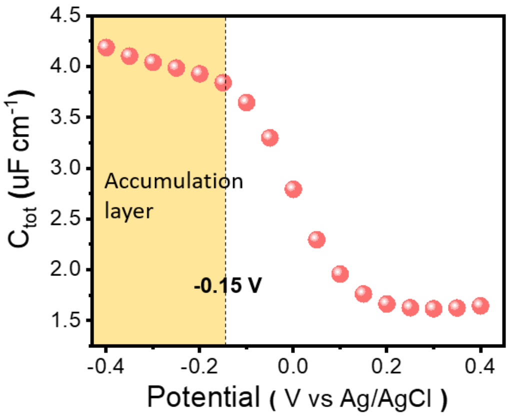

Fig. S8. Capacitance-voltage curve for ML MoS_2 transferred to Au by PMMA method (0.1 M K_2SO_4; 1kHz).

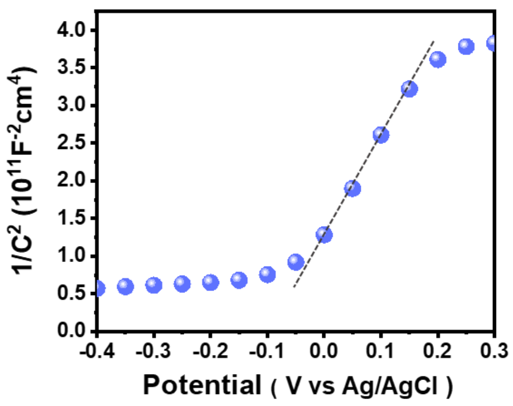

Fig. S9. Mott-Schottky plot of ML MoS_2 transferred to Au by PMMA method. The flat-band potential (V_{FB}) is approximately equal to -0.07 V (0.1 M K_2SO_4; 1kHz).

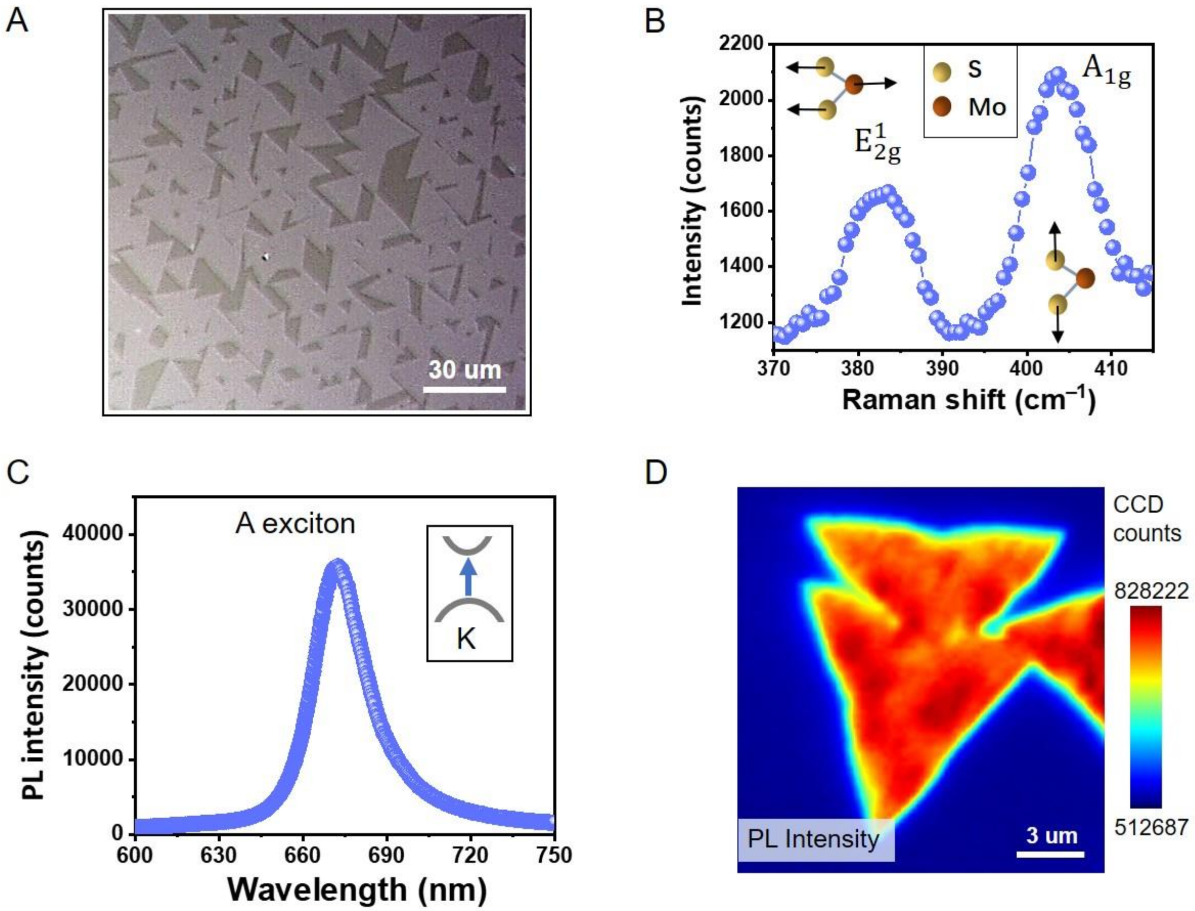

Fig. S10. Optical image and spectral characterization of CVD-grown ML MoS_2 on sapphire substrate. Optical image (A), Raman spectroscopy (B), PL spectra (C) and scanning PL maps (D) of ML MoS_2.

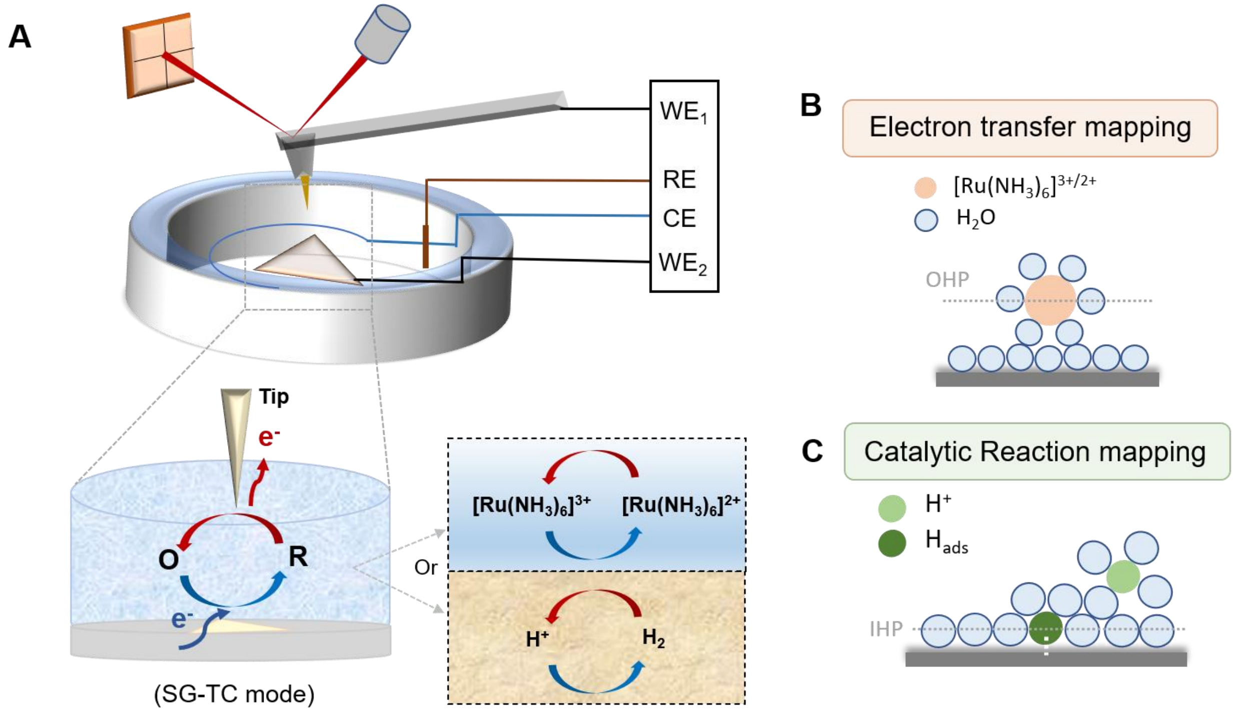

Fig. S11. *In-situ* electrochemical imaging characterization methods. (**A**) Schematic of AFM-SECM setup. SECM tip and electrocatalyst substrate were used as dual-working electrodes. Graphite and Ag/AgCl (saturated KCl) were used as counter electrodes and reference electrodes, respectively. Electroactive species are generated on a negatively biased substrate ($O + e^- \rightarrow R$) and collected by a positively biased tip ($R - e^- \rightarrow O$), and then anodic currents through the tip (i_{Tip}) are detected. (**B**) The reaction $[Ru(NH_3)_6]^{3+} + e^- \rightleftharpoons [Ru(NH_3)_6]^{2+}$ occurs at the outer Helmholtz plane (OHP), providing information on electron transfer (ET), therefore the redox pairs ($[Ru(NH_3)_6]^{3+/2+}$) were used to detect the ET sites distribution of MoS_2. (**C**) The HER occurs at the inner Helmholtz plane (IHP). The active sites where HER occurs are both ET sites and H^+ adsorption sites. Compared with the distribution of ET sites of MoS_2, the chemical sites (H^+ adsorption) distribution can be obtained.

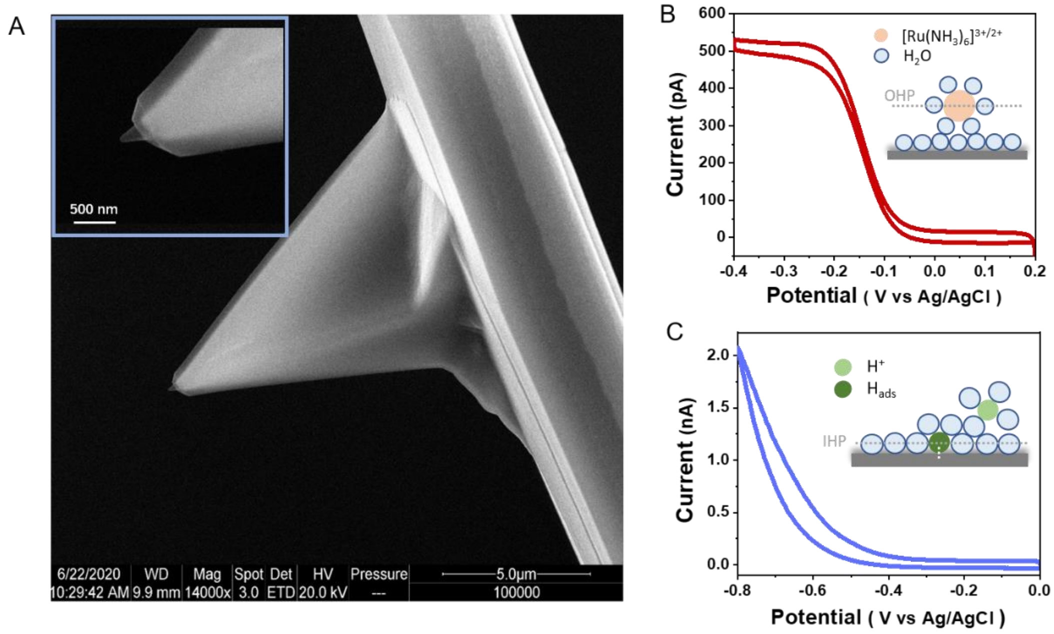

Fig. S12. SEM images and Cyclic voltammetry curves of PeakForce SECM probe. (A) SEM images of an Pt tip. The Pt probe is a cone-shaped microelectrode with a tip diameter of approximate 50 nm. Cyclic voltammetry curve of the SECM tip in 5 mM $[Ru(NH_3)_6]Cl_3$ + 0.1 M KCl. It shows a well-defined sigmoidal shape (B); Cyclic voltammetry curve of the SECM tip in 10 mM $HClO_4$ + 0.1 M $NaClO_4$ (C).

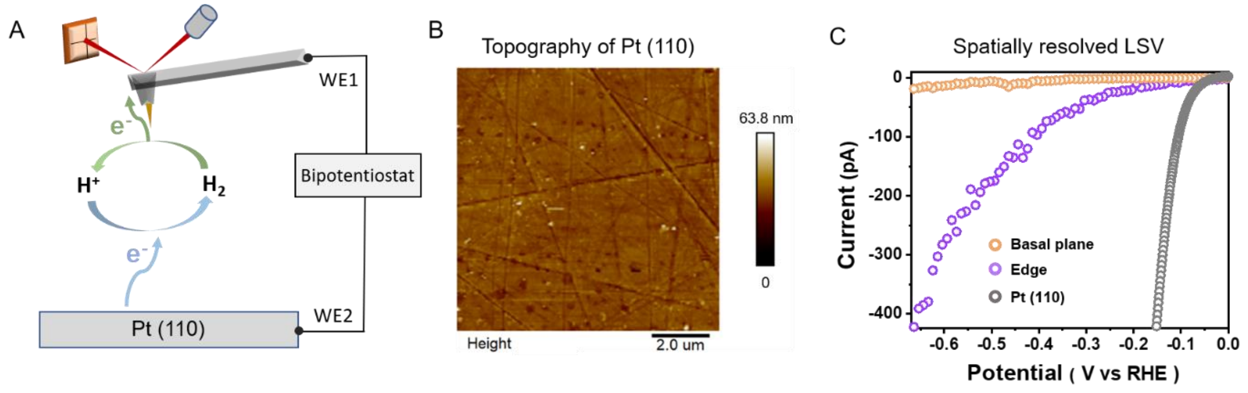

Fig. S13. Spatial resolved LSV curve of Pt (110). (A) Schematic diagram of spatially resolved LSV experimental set-up. The tip was biased at 0.3 V (vs Ag/AgCl) at a fixed height of h = 25 nm, while LSV was performed on the substrate (v = 5 mV/s). The solution contains10 mM $HClO_4$ and 0.1 M $NaClO_4$. (B) The topography of Pt (110). (C) Spatially resolved LSV curves of the edge and basal plane of ML MoS_2 and Pt (110). These LSV curves of the edge and basal plane of ML MoS_2 are also shown in Figure 4C in the main text.

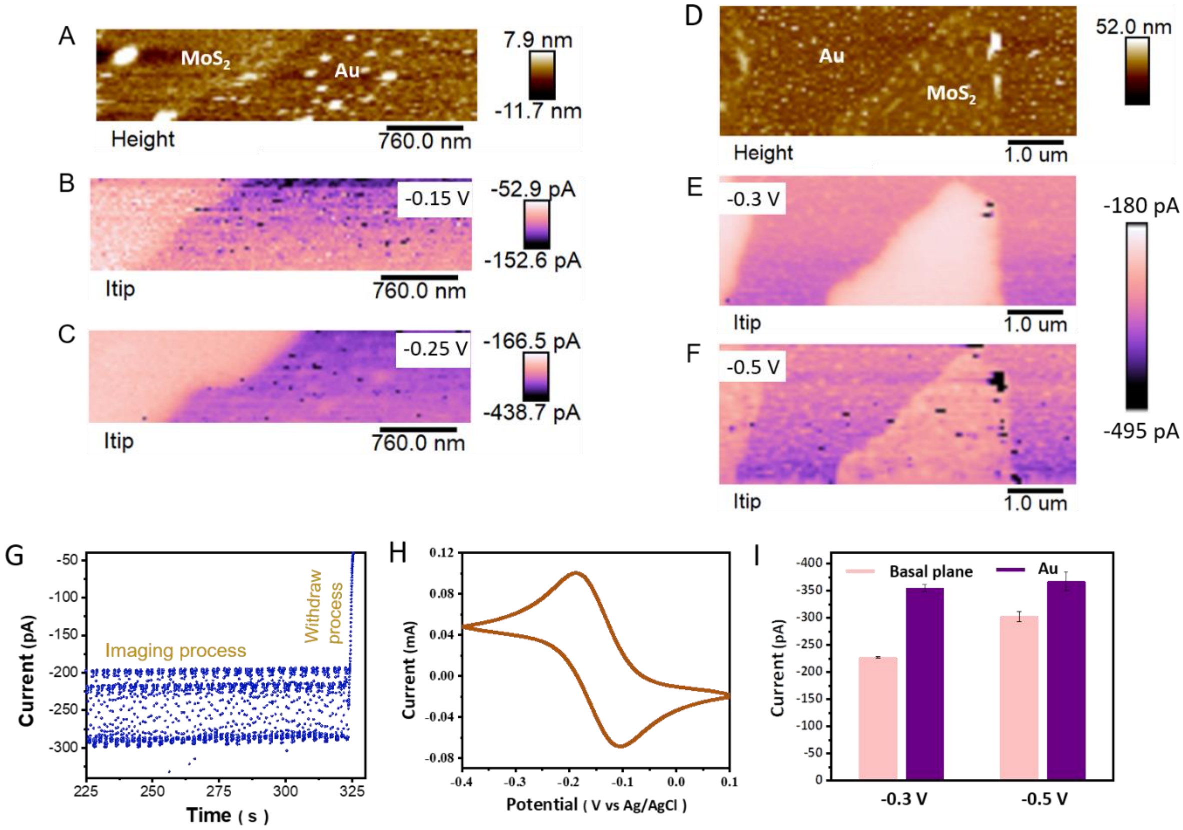

Fig. S14. ET maps of ML MoS_2 on Au substrate. (A-F) ET maps of ML MoS_2. 2D topography map (A and D) and ET current maps (B, C, E, F) (fixed height h = 25 nm, E_{Tip} = 0.3 V, E_{sub} = -0.15 V (B), -0.25 V (C), -0.3 V (E), -0.5 V (F) (vs Ag/AgCl), 5 mM $[Ru(NH_3)_6]Cl_3$ and 0.1 M KCl). Due to the large ET activity of Au, the ET current of the edge is covered by large background current. (G) Real-time imaging currents recorded by an electrochemical workstation. (H) Cyclic voltammograms on ML MoS_2-Au system in 5 mM $[Ru(NH_3)_6]Cl_3$ + 0.1 M KCl. The scan rate was set to 15 mV/s. (I) ET currents histogram of Au and the basal plane of ML MoS_2.

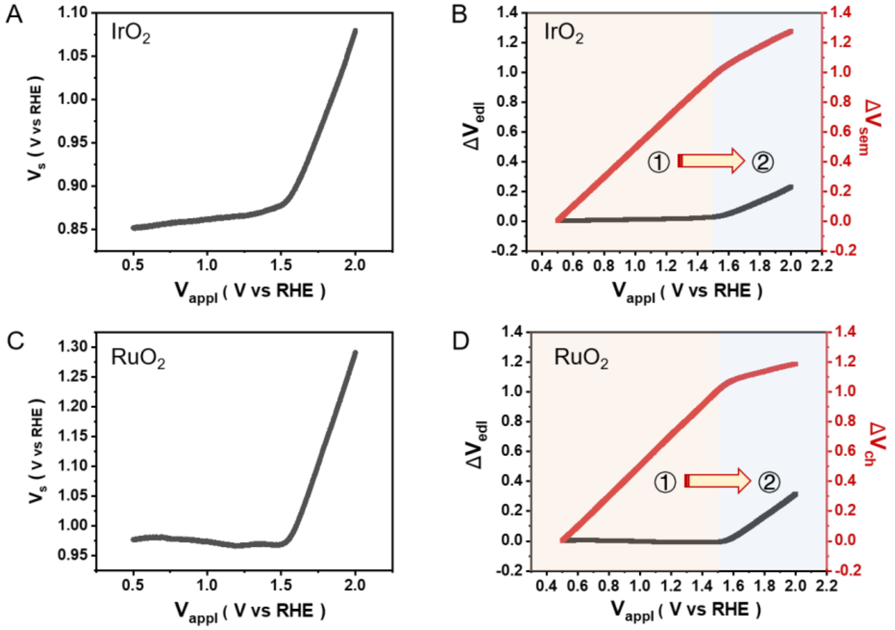

Fig. S15. Surface potential measurements of iridium oxide (IrO_2) and ruthenium oxide (RuO_2). (A) Surface potential measurement of IrO_2. (B) ΔV_{sem} and ΔV_{edl} of IrO_2 as a function of applied voltage. (C) Surface potential measurement of RuO_2. (D) ΔV_{sem} and ΔV_{edl} of RuO_2 as a function of applied voltage. Sulfuric acid solution with a pH of 1.81 was used as test solution.

REFERENCES

(1) Nie, W.; Zhu, Q.; Gao, Y.; Wang, Z.; Liu, Y.; Wang, X.; Chen, R.; Fan, F.; Li, C. Visualizing the Spatial Heterogeneity of Electron Transfer on a Metallic Nanoplate Prism. *Nano Lett.* **2021**, *21* (20), 8901–8909.

(2) Kresse, G.; Furthmüller, J. Efficiency of Ab-Initio Total Energy Calculations for Metals and Semiconductors Using a Plane-Wave Basis Set. *Computational Materials Science* **1996**, *6* (1), 15–50.

(3) Kresse, G.; Furthmüller, J. Efficient Iterative Schemes for *Ab Initio* Total-Energy Calculations Using a Plane-Wave Basis Set. *Phys. Rev. B* **1996**, *54* (16), 11169–11186.

(4) Blöchl, P. E. Projector Augmented-Wave Method. *Phys. Rev. B* **1994**, *50* (24), 17953–17979.

(5) Perdew, J. P.; Burke, K.; Ernzerhof, M. Generalized Gradient Approximation Made Simple. *Phys. Rev. Lett.* **1996**, *77* (18), 3865–3868.

(6) Perdew, J. P.; Ernzerhof, M.; Burke, K. Rationale for Mixing Exact Exchange with Density Functional Approximations. *The Journal of Chemical Physics* **1996**, *105* (22), 9982–9985.

(7) Huang, Y.; Nielsen, R. J.; Goddard, W. A.; Soriaga, M. P. The Reaction Mechanism with Free Energy Barriers for Electrochemical Dihydrogen Evolution on MoS_2. *J. Am. Chem. Soc.* **2015**, *137* (20), 6692–6698.

(8) Nørskov, J. K.; Bligaard, T.; Logadottir, A.; Kitchin, J. R.; Chen, J. G.; Pandelov, S.; Stimming, U. Trends in the Exchange Current for Hydrogen Evolution. *J. Electrochem. Soc.* **2005**, *152* (3), J23.
